# Cardiovascular diseases and Type 2 Diabetes in Bangladesh: A systematic review and meta-analysis of studies between 1995 and 2010

**DOI:** 10.1186/1471-2458-12-434

**Published:** 2012-06-13

**Authors:** Nazmus Saquib, Juliann Saquib, Tahmeed Ahmed, Masuma Akter Khanam, Mark R Cullen

**Affiliations:** 1 School of Medicine, Stanford University, Stanford, USA; 2 Clinical Sciences Division, International Center on Diarrheal Diseases and Research (ICDDR,B), Dhaka, Bangladesh

## Abstract

**Background:**

Belief is that chronic disease prevalence is rising in Bangladesh since death from them has increased. We reviewed published cardiovascular (CVD) and Type 2 Diabetes Mellitus (T2DM) studies between 1995 and 2010 and conducted a meta-analysis of disease prevalence.

**Methods:**

A systematic search of CVD and T2DM studies yielded 29 eligible studies (outcome: CVD only = 12, T2DM only = 9, both = 8). Hypertension (HTN) was the primary outcome of CVD studies. HTN and T2DM were defined with objective measures and standard cut-off values. We assessed the study quality based on sampling frame, sample size, and disease evaluation. Random effects models calculated pooled disease prevalence (95% confidence interval) in studies with general population samples (n = 22).

**Results:**

The pooled HTN and T2DM prevalence were 13.7% (12.1%–15.3%) and 6.7% (4.9%–8.6%), respectively. Both diseases exhibited a secular trend by 5-year intervals between 1995 and 2010 (HTN = 11.0%, 12.8%, 15.3%, T2DM = 3.8%, 5.3%, 9.0%). HTN was higher in females (M vs. F: 12.8% vs.16.1%) but T2DM was higher in males (M vs. F: 7.0% vs. 6.2%) (non-significant). Both HTN and T2DM were higher in urban areas (urban vs. rural: 22.2% vs. 14.3% and 10.2% vs. 5.1% respectively) (non-significant). HTN was higher among elderly and among working professionals. Both HTN and T2DM were higher in ‘high- quality’ studies.

**Conclusions:**

There is evidence of a rising secular trend of HTN and T2DM prevalence in Bangladesh. Future research should focus on the evolving root causes, incidence, and prognosis of HTN and T2DM.

## Background

Bangladesh is going through an ‘unsmooth’ epidemiological transition. While the death rate from infectious diseases has been decreasing slowly – largely due to successful immunization programs against childhood infectious disease, widespread use of oral saline for diarrhea, improvement in sanitation, and the availability of inexpensive antibiotics – the mortality rate from chronic diseases has been increasing very rapidly. The International Center for Diarrheal Diseases in Bangladesh (ICDDR, B), which has maintained a long standing surveillance site in rural Bangladesh, reported that 8% of the total deaths in its surveillance area in 1986 were due to chronic diseases [1]. The proportion of death due to chronic diseases increased to 41% by 1996 and 68% by 2006. Further, the report made a mortality projection that the death rate from cardiovascular diseases (CVD) would be 4 times higher in 2010 and 21 times higher in 2025 compared to its corresponding rate in 2003[1]. Bangladesh has been experiencing rapid urbanization for the past several decades [2,3], which raises the concern that the chronic disease burden may be even higher for urban populations. Unplanned development in the urban areas has created an environment that is prohibitive and unsafe for physical activity [4]. Increased access to and popularity of fast food may also contribute to poorer diet quality, particularly among the city’s affluent class [5]. Further, high density living has contributed to increased air pollution in urban areas [6].

Besides the high-mortality rate from chronic diseases, Bangladeshis are developing these diseases at an earlier age than in the past. For example, the Bangladesh Institute of Research and Rehabilitation for Diabetes, Endocrine, and Metabolic Disorders (BIRDEM) patient registry showed that the mean age at diagnosis for Type 2 Diabetes (T2DM) was significantly lower in 2005 than in 1995 [7]. Similarly, the INTERHEART Study- a global case–control study of risk factors for acute myocardial infarction (MI)- reported that the mean age of MI among the Bangladeshis (51.9 years) was 6 years lower than the non-South Asians (58.8) and the lowest among all South Asians [8]. An analysis of the INTERHEART control participants further showed that the Bangladeshis had the highest prevalence of CVD risk factors among the participating countries: current and former smoking (59.9%), abdominal obesity (43.3%), self-reported history of hypertension (14.3%), depression (43%), and elevated ApoB_100_/Apo-I ratio (59.6%). Bangladeshis had the lowest prevalence for regular physical activity (1.3%) and daily intake of fruits and vegetables (8.6%) [8].

The rising chronic disease burden and high prevalence of risk factors for these diseases among Bangladeshis make it imperative that the country undertakes appropriate measures to address these problems. A recent review assessed the ongoing chronic disease programs (n = 11) in Bangladesh to indentify key priorities for the country [9]. It concluded that these programs, both governmental and non-governmental, partially met five of the six chronic disease prevention strategies, which were developed by the World Health Organization (WHO) and adopted by the Bangladesh government. The only strategy that had not been met by any of the programs was the ‘prevention and control of chronic diseases through the promotion of research’ [9].

If research is to guide future prevention strategies, the first step is to gather the existing evidence. To this end, we compiled the results of all research studies on CVD and T2DM, published in national and international journals, between 1995 and 2010. We aimed to assess the prevalence/incidence of diseases and the associated risk factors reported in these studies. We examined the study attributes, sampling strategies, participants’ characteristics, and strata-specific disease estimates. The specific review objective were: (1) to determine the extent of research that has been done for each disease, (2) to identify populations who are at a higher risk of disease, and (3) to recommend the future direction of prevention research efforts.

## Methods

### Search strategy

We systematically explored both the international (PubMed) and country specific (BanglaJol) search engines to locate the relevant articles. We categorized the search terms according to location, methodology and outcomes: (1) Location: “Bangladesh.” (2) Method: “prevalence, cross-sectional, cohort studies, survey.” (3) Outcome: “diabetes, diabetes mellitus, non-insulin dependent diabetes mellitus, NIDDM, type 2 diabetes, cardiovascular disease, CVD, myocardial infarction, ischemic heart disease, hypertension, high blood pressure, coronary artery disease”. The “AND” Boolean operator was used to combine search terms across the categories and the “OR” was used to combine within the categories. Further, we limited the search to studies that only involved human participants and were published between January 1, 1995 and December 31, 2010. We screened the studies using the following inclusion criteria: (1) had prevalence or incidence data available on either CVD or T2DM, (2) had selected a sample from within Bangladesh, and (3) had published results between 1995 and 2010. Once we identified the eligible studies, we made further exclusions based on sample, study design, and publication type. We retrieved the full-text for the selected studies either via the Internet or from the author directly; however, full text could not be obtained for 5 studies. The prevalence data for these studies was extracted from the abstract.

### Exclusion of studies

**Figure 1:**
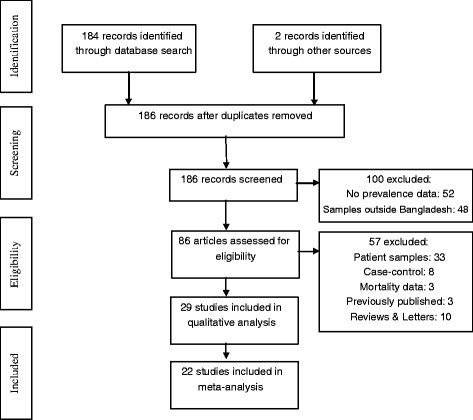
Consort diagram.

We screened 186 articles based on our search results and 86 of them met our inclusion criteria. Of the 100 ineligible studies, 52 were excluded because they did not report an estimate of disease prevalence and 48 were excluded because the samples did not involve Bangladeshis or included only those living in other countries. From the 86 eligible studies, we excluded an additional 57 studies for the following reasons: (1) had a sample of only patients (n = 30) or had calculated prevalence from the hospital outpatient department (n = 3), (2) had a case–control design (n = 8), (3) had only mortality data (n = 3), (4) had data that was published previously (n = 3), or (5) had a review or letter format (n = 10). The remaining 29 studies [10-38], were the focus of this review (Figure 1). We specified ‘general population sample’ as the criterion for inclusion in the meta-analysis. A total of 22 studies were used to calculate the pooled prevalence. We analyzed the remaining 7 studies, which sampled special populations (elderly, pregnant, or professional), separately.

### Data extraction

We extracted the following information for each reviewed study: (1) Authors and publication year, (2) Title and journal, (3) Study location (urban or rural), (4), Study design, (5) Sampling strategy (random or non-random), (6) Sample size, (7) Sample characteristics such as age and gender, (8) Disease type: CVD [Hypertension (HTN), Ischemic Heart Disease (IHD)] and T2DM (9) Diagnostic criteria for each disease, (9) Outcome assessment (objective or subjective), (10) Prevalence (overall and gender- or location-specific) for each disease, (11) Risk factors significantly associated with each disease, and (12) Prevalence of conditions indicating risk for T2DM: Fasting plasma glucose (FPG), and Impaired glucose tolerance (IGT).

### Quality assessment of studies

We chose three distinct study characteristics to assess the quality of the reviewed studies. Each study was evaluated whether it used a random sample (yes = 1, no = 0), whether it had a sufficient sample size (yes = 1, no = 0), and whether it assessed disease outcome with an objective measure (yes = 1, no = 0). The cut-off for a sufficient sample size was set at 500 participants. With this scoring any study could tally as high as 3 and as low as 0. Studies that received 3 points were labeled as ‘high quality’, 2 points as ‘moderate quality’, and 1 point or less as ‘poor quality’.

### Analysis plan

We marked the location of the reviewed studies on a country map, tabulated the study attributes separately by the disease (Additional file 1: Table S1: CVD, Additional file 2: Table file S2: T2DM), and coded the estimate for disease prevalence (HTN, IHD, T2DM). A number of CVD studies, with HTN as an outcome, did not report an overall HTN prevalence, but rather reported systolic and diastolic HTN separately. For these studies, we used their diastolic HTN values, which were more conservative than their corresponding systolic HTN values, to calculate the overall HTN prevalence.

We conducted meta-analysis to report the prevalence of diseases (HTN, T2DM). The methodology that we followed for the meta-analysis was described in details by Neyeloff et al. [39]. Briefly, for each study, we calculated the following variables: 1) standard error of the prevalence, 2) variance, 3) study weights (inversed variance), 4) study weight*prevalence estimate, 5) study weight* (prevalence estimate)^2^ and 6) (study weight)^2^. We used these variables to estimate Q (measure of heterogeneity among studies) and I^2^ (percent between-studies variability). Based on Q and I^2^ values, we chose random effects models to report pooled prevalence estimates (HTN, T2DM) and the associated 95% confidence interval (CI). We followed the same procedures to determine the stratum-specific pooled prevalence: gender (male vs. female), study location (urban vs. rural), study quality (high, moderate, or poor), and time period (1995–2000, 2001–2005, 2006–2010). In addition, we reported pooled prevalence data on FPG and IGT.

Finally, we extracted HTN and T2DM-specific covariate data. We extracted data on life-style related risk factors of chronic diseases such as smoking, obesity, physical activity, fruits and vegetable consumption, and referenced them in the discussion section.

## Results

### General characteristics of the reviewed studies (n = 29)

**Figure 2:**
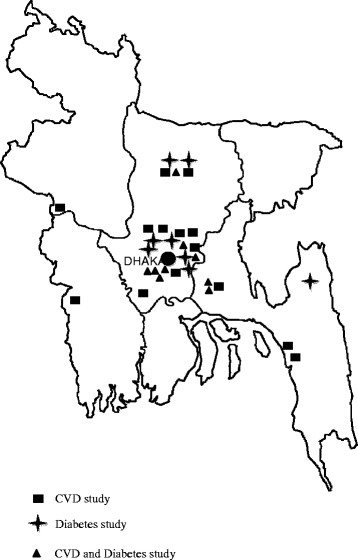
Map of Bangladesh: Study locations.

Most studies were conducted in two out of the six divisions in the country. The greatest concentration of studies has been in the surrounding areas of the capital city of Dhaka (Figure 2). Twenty seven of the 29 studies, which reported prevalence data (CVD and/or T2DM), had a cross-sectional design. The remaining two studies were a prospective cohort and a randomized trial.

Over fifty percent of studies (n = 15) selected participants with a random procedure, implemented at one of three levels: village/city ward, household, or individual. Another eight studies enrolled everyone who resided in a chosen geographic area and met the study eligibility criteria; for example, all who were eligible in a village (or group of villages). Three studies used a non-random sample and information on the sampling strategy could not be ascertained for the remaining three studies. Seventy-five percent of the studies (n = 22) selected the sample from the general population. The lower age limit for eligibility was set to 20 in most of these studies, except in two where it was 13 and 15, respectively. The sample of the remaining seven studies came from older adults (age > 50, n = 3), pregnant women (n = 2), and working professionals (n = 2). Twenty-five studies had both male and female participants, 3 studies had only women, and one did not provide gender information. Sixteen studies were conducted in rural areas (55%), 8 in urban areas (28%), and the remaining 5 studies had both urban and rural samples (17%).

According to the criteria that we used to rate the studies, 11 were of ‘high quality’ (38%), 12 were of ‘moderate quality’ (41%), and the remaining 6 were of ‘poor quality’ (21%).

### Outcome assessment and definitions

Cardiovascular disease (CVD) was the sole outcome in 12 studies, T2DM alone in 9 studies, and both in the remaining 8 studies. Twenty-five studies used objective measures to assess disease status, one used self-report data, and three studies did not provide adequate information.

The definition of HTN varied across studies: (a) systolic blood pressure (SBP) ≥ 140 mmHg or diastolic blood pressure (DBP) ≥ 90 mmHg (n = 6), (b) SBP ≥ 140 and/or DBP ≥ 90 and/or anti-hypertensive medication use (n = 4), (c) SBP ≥ 140 and DBP ≥ 90 (n = 1), (d) SBP ≥ 140 (n = 1), (e) DBP ≥ 90 or medication use (n = 1), (f) SBP ≥ 135 or DBP ≥ 85 (n = 2), (g) self-reported (n = 1), (h) not specified (n = 4).

All three studies determined IHD through the use of electrocardiogram. However, only one study provided criteria (the presence of Q wave) as to how it defined IHD.

Most studies defined T2DM according to the World Health Organization, WHO-1980, the WHO-1999, or the American Diabetes Association, ADA-1997 classification system [40,41]. The definition of T2DM varied according to whether the test was either a fasting or 2-hr post glucose and whether the specimen was either blood or plasma. T2DM was self-reported in one study and not defined in another study. One study had gestational diabetes mellitus (GDM) as its outcome.

### Prevalence estimates

**Figure 3:**
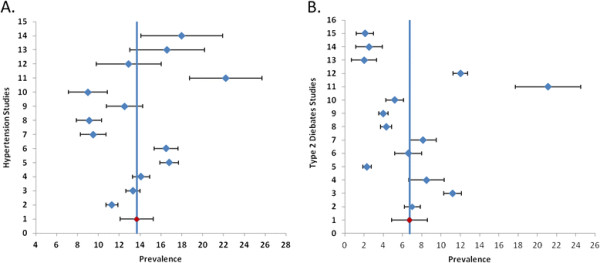
**Forest Plot of (A) Hypertension (HTN) [ **[10]**-**[12]**,**[14]**,**[15]**,**[18]**,**[20]**,**[23]**-**[26]**,**[37]**,**[38]**] and (B) Type 2 diabetes mellitus (T2DM) [ **[10]**,**[14]**,**[16]**,**[20]**,**[22]**,**[24]**,**[25]**,**[30-34][35]**-**[36]**] studies published between 1995 and 2010.** All studies (HTN = 13, T2DM = 14) selected samples from the general adult population. Pooled estimates (red) and 95% CI were calculated with random effect models.

**Figure 4:**
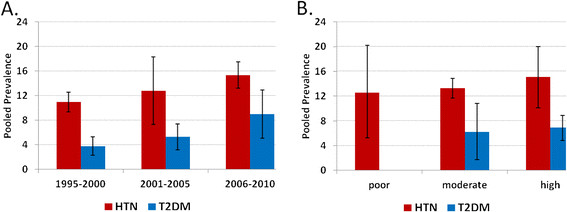
**Pooled estimates of Hypertension (HTN) and Type 2 diabetes mellitus (T2DM) by (A) 5-year intervals: 1995–2000, 2001–2005, and 2006–2010 and (B) study quality: poor, moderate, and high. **All studies (HTN = 13, T2DM = 14) selected samples from the general adult population. Estimates and 95% CI were calculated with random effect models.

*HTN (n = 20):* (1) General population sample (n = 13): Overall HTN prevalence estimates ranged from 9.0% to 22.2% (median = 13.3%). The pooled HTN prevalence was 13.7% (95%CI: 12.1%–15.3%, I^2^: 95%) (Figure 3, A). The pooled prevalence within strata was: 12.8% (95%CI: 10.8%–14.7%) for males, 16.1% (95%CI: 13.2%–19.0%) for females, and 14.3% (95%CI: 12.7%–15.8%) for rural areas. There was one study in the urban area and the prevalence was 22.2% (95%CI: 18.7%–25.7%). The pooled HTN prevalence within each 5-year time period was: 1995–2000 = 11.0% (95%CI: 9.4%–12.6%), 2001–2005 = 12.8% (95%CI: 7.3%–18.3%), and 2006–2010 = 15.3% (95%CI: 13.2%–17.5%); and within each category of study quality was: poor = 12.6% (95%CI: 5.3%–20.0%), moderate = 13.3% (95%CI: 11.7%–14.9%), and high = 15.1% (95%CI: 10.1%–20.0%) (Figure 4). The between-study variability (I^2^) in the strata-specific meta-analyses ranged from 74% to 97%. (2) Special population sample (n = 7): HTN prevalence ranged between 16.8% and 64% in 3 studies of older adults. The prevalence was 5.4% and 9.2% respectively in 2 studies of pregnant women, and 16.6% and 17.3% respectively in 2 studies of working professionals (government employee and health care providers).

*IHD (n = 3*): IHD prevalence was between 2.7% and 3.4% in two studies with a rural sample and 19.6% with an urban sample of working professionals.

*T2DM (n = 17)*: (1) General population sample (n = 14): Overall T2DM prevalence estimates ranged from 2.0% to 21.1% (median = 5.9%). The pooled T2DM prevalence was 6.7% (95%CI: 4.9%–8.6%, I^2^: 98%) (Figure 3, B). The pooled prevalence within strata was: 7.0% (95%CI: 5.1%–8.9%) for males, 6.2% (95%CI: 4.2%–8.1%) for females, 5.1% (95%CI: 2.9%–7.3%) for rural areas, and 10.2% (95%CI: 6.0%–14.4%) for urban areas. The pooled T2DM prevalence within each 5-year time period was: 1995–2000 = 3.8% (95%CI: 2.3%–5.3%), 2001–2005 = 5.3% (95%CI: 3.2%–7.4%), and 2006–2010 = 9.0% (95%CI: 5.1%–12.9%); and within each category of study quality was: moderate = 6.2% (95%CI: 1.7%–10.8%), and high = 6.9% (95%CI: 4.8%–8.9%) (Figure 4). The between-study variability (I^2^) in the strata-specific meta-analyses ranged from 91% to 99%. (2) Special population sample (n = 3): T2DM prevalence was 6.8% in one study of pregnant women, and ranged between 4.5% and 12.3% in two studies of working professionals.

### Covariate assessment

Nine studies reported data on covariates of HTN. Being female, older, obese, diabetic, having a higher education, or from a higher socioeconomic status were significantly associated with a higher prevalence of HTN. Additionally, urban dwelling was reported to be associated with a higher prevalence of HTN in one study and with a lower prevalence in another study. Further, the studies that were conducted in arsenic contaminated areas reported a dose–response relationship between arsenic exposure and HTN; the prevalence of HTN increased incrementally across the exposure categories.

Ten studies reported T2DM-related covariate data. Being male, older, obese (both general and central), hypertensive, having a sedentary lifestyle, having a family history of diabetes, and a higher socioeconomic status were significantly associated with higher prevalence of diabetes.

## Discussion

### Secular trend

The meta-analyses of the prevalence data suggest a rising secular trend of chronic disease in Bangladesh. For both HTN and T2DM, the pooled prevalence estimates were lowest during 1995–2000, highest during 2006–2010, and in-between during 2001–2005 (Figure 4). Compared with the estimates during 1995–2000, those during 2001–2005 were non-significantly higher and those during 2006–2010 were significantly higher. This supports the ‘epidemiological disease shift’ reported previously, which showed an increase in death due to chronic diseases with a concomitant decrease in death due to infectious diseases [1]. The rising trend of HTN and T2DM could be explained, in part, by the increased life expectancy of the Bangladeshi population as well as the increased presence of known risk factors for chronic diseases. Between 1995 and 2010, the mean life expectancy has risen by approximately 6 years (62.1 to 68.3 years) [42]. Also, Bangladeshis rank highest among South Asians in terms of tobacco use, physical inactivity, and poor dietary habits [8]. We anticipated that methodological differences such as sample selection and diagnostic criteria across the studies could influence the estimate of the secular trend. Therefore, we limited our analysis to only those studies that selected a sample from the general population. Further, the cut-off value of systolic (140 mmHg) and diastolic (90 mmHg) blood pressure used to define HTN did not change during the review period. We acknowledge that the diagnostic criteria [43,44] for T2DM have become less stringent during this time period but this small change alone is unlikely to explain the two-fold rise in the T2DM prevalence.

### Disease comparison

The HTN prevalence that we found in this review is comparable to the HTN prevalence reported in the other South-Asian Countries, [45-53]. Although a formal comparison among these countries does not fall within the scope of this review, it is worthwhile to compare the data between Bangladesh and India, as they are geographically contiguous, share common traits in culture, and have similar life expectancy and chronic disease risk factor profiles [8,42]. For example, the HTN prevalence in the rural (pooled: 14.3%) and urban (single study: 22.2%) Bangladesh is well within the range of prevalence estimates for rural (12%–17%) and urban (20%–40%) India [46]. Similarly, the IHD prevalence in the rural areas is comparable between the two countries (Bangladesh = 2.7%–3.4% vs. India = 3%–4%) [12,20,21,48,54]. The estimates for the urban areas cannot be compared because the Bangladesh estimate (19.6%) comes from only one study, which selected a small sample from a special population (government employees) [27].

The rising HTN prevalence in Bangladesh is worrisome since a large segment of the population is unaware that they have HTN and since many HTN patients do not have their blood pressure properly managed with medication. Between fifty and eighty-five percent of the participants who were diagnosed with HTN did not know they had HTN prior to the study assessment [55]. Although allopathic anti-HTN medication use (97%) is the norm among those who were diagnosed with HTN, only a quarter of them were found to have their blood pressure controlled with medication. Since uncontrolled HTN is a major risk factor for myocardial infarction (MI), it may partly explain why Bangladeshis have the lowest mean age for the development of MI among the nations of the world [8].

Similar to HTN and IHD, the T2DM prevalence in Bangladesh is also comparable to the corresponding estimates from other South-Asian countries such as India [45]. Although, in both countries, the prevalence is low in the rural areas (Bangladesh = 5.1%, India = 3.8%), it is considerably higher in the urban areas (Bangladesh = 10.2%, India = 11.8%) [45]. At the same time a significant portion of the population have conditions such as IFG or IGT, and therefore are at risk of developing T2DM [14,30,35]. For example, in our reviewed studies, the pooled prevalence for IFG and IGT were found 7.0% (95%CI: 3.2%–10.8%, I^2^ = 99%) and 8.2% (95%CI: 3.9%–12.4%, I^2^ = 99%), respectively [10,16,22,25,30,31,33,35,36].

Of all the factors that may be contributing to the rise of HTN and T2DM in Bangladesh, behavioral risk factors are likely to be the most salient. A recent review suggests that 90% of the chronic diseases among the South-Asians are attributable to life-style factors [54]. Survey-data indicates that between 47% and 63% of men and less than 3% of women in Bangladesh are daily smokers [14,55-57]. A significant portion of these smokers uses filter-less cigarettes. Further, smoke-less tobacco use (with bettle-leaf or chewed/sniffed in the powdered form) is quite common (30%) in the population, particularly among the poor and in rural areas, irrespective of gender [57]. Bangladeshis also rank highest among the South Asians in terms of physical inactivity and low intake of fruits and vegetables [58,59]. And, although the overweight prevalence is relatively low (men = 10%, women = 15%), a large portion of Bangladeshis are, in fact, centrally obese (men = 25%, women = 60%) [34,35,60].

### Strengths and limitations of the reviewed studies

It is notable that dozens of studies have been conducted in the last fifteen years to assess the risk factors and overall burden of chronic disease in Bangladesh, despite it being one of the poorest countries in the world and having little allocation of government funding for research. Many adopted a unique sampling strategy, in which they selected a village or a group of villages and enrolled all eligible participants. This particular strategy ensured that each study, besides having a robust sample, also had adequate representation in age, gender, and socioeconomic strata. Studies were also conducted in special populations such as pregnant women [17], health care professionals [28], government employees [27], and slum dwellers [32]. Inclusion of both urban and rural samples enabled a few studies to make a direct comparison of disease prevalence and risk factors between these two groups [14,55]. Objective measurement (such as blood pressure measurement, echocardiogram, or blood analysis) of disease outcomes was common to most studies. Further, studies clearly defined the eligibility criteria and included relevant covariate data.

A general limitation of the reviewed studies was a lack of representative study samples in the urban areas. For example, the studies that were conducted in the capital have recruited participants either sharing a common living environment [32] (e.g. slum dwellers) or a common profession [28] (e.g. health care workers). Such samples are unlikely to provide a true estimate of CVD or T2DM prevalence for a city with 12–15 million residents living in a wide range of socioeconomic and environmental conditions. On a similar note, using a single or a group of villages in a certain geographic location may not capture the disease prevalence for a country with approximately 68 thousand villages.

### Strengths and limitations of the review

By categorizing the studies according to the markers of good study design (random sample, sufficient sample size, objective measure), we showed that the disease prevalence varied by the study quality. The prevalence was generally higher in ‘high-quality’ studies than in ‘poor-quality studies’, for both HTN and T2DM. We conducted meta-analyses to calculate the pooled prevalence of disease. However, data interpretation should be done cautiously since the heterogeneity (between-study variability) was very high, despite the use of random effects models to accommodate this variability. A limitation is the small number of studies, which have prevented us from stratifying the time trend analysis by study quality.

A general difficulty of this project was to gather all the published data on CVD and T2DM for the review period, since not all Bangladeshi journals are available electronically, and those that have electronic text came into existence recently. Although we were able to collect a number of full text articles that were otherwise unavailable in the Web through personal communications with Bangladeshi researchers, we had to rely on the abstract for a few studies. In most cases, the information needed for the quality rating was available in the abstract and in the two instances where it was missing, we have imputed with the lower (i.e. more conservative) value. There may also be unpublished disease prevalence data that we have missed in our review.

### Recommendations

Although an adequate number of HTN prevalence studies have been conducted, they were mostly done in and around Dhaka city, leaving many regions of the country unstudied. Little data exist on the prevalence of other cardiovascular diseases such as ischemic heart disease. Further, we did not find prevalence data on rheumatic heart disease or tuberculous pericardial disease, despite the fact that many Bangladeshis, especially the slum dwellers, suffer from poor nutrition and live in unsanitary conditions -- both of which have been associated with the development of these diseases. No study has reported to date the impact of nutritional (i.e. sodium, refined sugar intake), occupational, and various environmental factors (i.e. migration to the city, salination of drinking water, air pollution) on CVD or T2DM. It is important that these issues be explored for a number of reasons: (1) the urban population has been growing on an average of 3.5% a year [61], (2) food and drinking water in the coastal area has been greatly affected by a rise in salinity [62,63], and (3) the urban areas have seen a decline in air quality [6].

We recommend that future prevalence studies focus on the gaps identified in this review. In addition, we suggest that samples of the future studies are selected systematically, according to the administrative structure of the country (division, district, sub-district, etc.), in order to capture any differences in disease prevalence by geographical regions. We did not find any longitudinal cohort studies on CVD or T2DM. This is a significant gap in the knowledge and understanding of these chronic diseases in the context of Bangladesh. Such studies would provide essential information on the incidence of these diseases, their associated risk factors, and the groups that are at higher risk of developing them. Further, longitudinal data are necessary to understand disease progression and prognosis.

We recognize the potential challenges to conducting cohort studies in Bangladesh. Long-term follow-up of participants may be difficult. In the urban areas, many people are either renters or slum dwellers who change their residence frequently. A similar challenge may arise in rural areas as there is constant migration to the cities for employment. Other challenges include the absence of either disease/death registries or electronic medical records. We acknowledge that the development of a cohort study would require considerable effort and data would not be available for many years. In the meantime, retrospective cohort studies could be conducted using medical records available in various government (NICVD-National Institute of Cardiovascular Diseases) and private institutions (ICDDR,B-International Center on Diarrheal Diseases and Research, Bangladesh; BIRDEM-Bangladesh Institute of Research and Rehabilitation for Diabetes, Endocrine, and Metabolic Disorders; NHF-National Heart Foundation). Such retrospective studies could begin to address some of the research issues outlined above such as disease complications and treatment adherence.

## Conclusions

This review of the Bangladeshi research studies on CVD and T2DM between 1995 and 2010 concludes that the prevalence of HTN and T2DM in the population is significant and that there is evidence of a rising secular trend for these two diseases. The disease prevalence was higher in older adults, women, and those living in urban areas. Also, established risk factors for HTN and T2DM such as smoking and central obesity are already widespread in the population. Further, significant proportions of Bangladeshis are either physically inactive, inadequate in their intake of fruits and vegetables or both. Appropriate research could provide the foundation for developing effective policies and intervention strategies to address the growing epidemic of HTN and T2DM in Bangladesh.

## Competing interests

No author of this paper had any competing interests.

## Authors’ contributions

Dr. Nazmus Saquib, Dr. Juliann Saquib, and Dr. Mark Cullen of Stanford were involved in all aspects of the paper including conception and design of the study, acquisition, analysis and interpretation of data, drafting and revising the manuscript and approval of the final version. Doctors Masuma Khanam and Tahmeed Ahmed, collaborators from the ICDDR,B made substantive intellectual contributions to the interpretation of the data, draft of the manuscript and final version for publication. All authors read and approved the final manuscript.

## Pre-publication history

The pre-publication history for this paper can be accessed here:

http://www.biomedcentral.com/1471-2458/12/434/prepub

## Supplementary Material

Additional file 1 **Table S1.**Cardiovascular diseases prevalence in Bangladesh: A summary of epidemiological studies published between 1995 and 2010. Click here for file

Additional file 2 **Table S2.**Type 2 diabetes (T2DM) prevalence in Bangladesh: A summary of epidemiological studies published between1995 and 2010. Click here for file
