# Supplementary material for: Cardiovascular diseases and Type 2 Diabetes in Bangladesh: A systematic review and meta-analysis of studies between 1995 and 2010
Source: BMC Public Health. 2012 Jun 13;12:434. doi: 10.1186/1471-2458-12-434 (PMC3487781; doi:10.1186/1471-2458-12-434)
Supplement: Additional file 1 — Table S1.Cardiovascular diseases prevalence in Bangladesh: A summary of epidemiological studies published between 1995 and 2010. [file 1471-2458-12-434-S1.doc]

**Additional Table 1** Cardiovascular diseases prevalence in Bangladesh: A summary of epidemiological studies published between 1995 and 2010.

| **Authors/Year** | **Diagnostic criteria** | **Sample Size** | **Sample Characteristics** | **Prevalence** | **Prevalence by Strata** | **Significant Risk Factors** |
| --- | --- | --- | --- | --- | --- | --- |
| Sayeed MA, 1995 [10] | SBP≥ 140 OR DBP > 90 | 1005 | rural  age > 15 | sHTN=10.5%, dHTN=9.0% | not reported | age, BMI |
| Rahman M, 1999 [11] | SBP≥ 140 AND DBP≥ 90 | 1595 | rural  age ≥ 30 | HTN = 12.5% | male =12.3%  female = 13.7% | arsenic exposure |
| Zaman MM, 1999 [12] | DBP≥ 90 or medication | 13,288 | urban/rural  urban age ≥ 18  rural:  sample 1: age ≥ 20  sample 2: age ≥ 15 | HTN = 11.3% | urban = 13.3%. Rural:  sample 1=7.8%  sample 2= 9.0% | not reported |
| Hypertensive study Group, 2001 [13] | SBP≥ 140 and/or DBP≥ 90 and/or medication | 480 | urban/rural  age ≥ 60 | HTN = 64% | urban = 75%  rural = 53% | BMI, education, diabetes, urban dwelling |
| Zaman MM, 2001 [14] | SBP≥ 140 and/or DBP≥ 90 and/or medication | 510 | rural  age ≥ 18 | HTN = 12.9% | male = 9.8%, female=15.6%* | not reported |
| Sayeed MA, 2002 [15] | unknown | 2361 | urban/rural  age ≥ 20 | sHTN = 14.4% dHTN = 9.1% | unknown | age, BMI, rural area, and upper-class |
| Sayeed MA, 2003 [37] | SBP≥ 140 | 4923 | rural  age ≥ 20 | HTN =16.5% | male = 13.5%, female=19.2%* | sex, waist-to-height ratio |
| Sayeed MA, 2005 [17] | SBP≥ 140 or DBP≥ 90 | 147 | rural pregnant women  age=18-44 | sHTN=6.8%, dHTN=5.4% | not applicable | not reported |
| Chen Yu, 2006 [18] | SBP≥ 140 or DBP≥ 90 or medication | 11,116 | rural  age ≥ 18 | HTN = 13.3% | male = 13.6%, female=13.0% | animal protein dietary pattern |
| Mamun AA, 2006 [19] | SBP > 140 or DBP > 90 | 1584 | urban pregnant women  mean age = 22 | HTN = 9.2% | not applicable | not reported |
| Zaman MM, 2007 [20] | IHD = pathological Q wave  HTN = not defined | 447 | rural age ≥ 20 | IHD =3.4%  HTN=18% | IHD:  male = 4.6%  female =2.7% | not reported |
| Ahmed, 2007 [21] | SBP≥ 135 orDBP≥ 85; IHD not defined | 226 | rural age ≥ 50 | IHD = 2.7%  sHTN =17.7%  dHTN=16.8% | male:  sHTN=15.7%  female:  sHTN=20.0% | not reported |
| Sayeed MA, 2007 [38] | SBP≥ 140 or  DBP≥ 90 | 2384 | urban/rural  age ≥ 20 | sHTN=14.3%, dHTN=9.5% | male:  sHTN=13.3%  dHTN=10.1%  female:  sHTN=14.9%  dHTN=9.2% | age, weight, BMI, WHR, WHTR |
| Ahmed S, 2008 [23] | unknown | 501 | rural women only  age = 15 - 49 | HTN = 16.6% | not applicable | not reported |
| Van Minh H, 2008 [24] | self-reported | 8096 | rural  age= 25 -64 | HTN =14.1% | male = 9.3%  female = 19.0% | age, sex |
| Sayeed S, 2008 [25] | SBP≥ 135 or  DBP≥ 85 | 705 | urban  age ≥ 25 | sHTN=14.7%  dHTN=22.2% | male:  sHTN=15.9%  dHTN=27.5% female:  sHTN=14.1%  dHTN=19.5% | not reported |
| Van Minh H, 2009 [26] | SBP≥ 140 or  DBP≥ 90 | ~ 8000 | rural  age=25-64 | HTN = 9.3% -24.1% | Male:  7.4%-20.3%  Female:  11.2 - 27.4%, | age, sex, weight, education |
| Ahsan SA, 2009 [27] | SBP≥ 140 or DBP≥ 90; IHD:not defined | 163 | urban  mean age = 44.8 | IHD =19.6%  HTN = 16.6% | not reported | not reported |
| Das S, 2010 [28] | unknown | 1200 | urban | HTN = 17.3% | unknown | unknown |
| Moni Ma, 2010 [29] | SBP≥ 140 and DBP≥ 90 or medication | 317 | urban  mean age = 67.1 | HTN = 44.8% | unknown | Unknown |

SBP: systolic blood pressure, DBP: diastolic blood pressure, HTN: hypertension, sHTN: systolic hypertension, dHTN: diastolic hypertension, BMI: body mass index, IHD: ischemic heart disease, WHR: Waist to hip ratio, WHTR: Waist to height ratio.
