# Supplementary material for: Cardiovascular diseases and Type 2 Diabetes in Bangladesh: A systematic review and meta-analysis of studies between 1995 and 2010
Source: BMC Public Health. 2012 Jun 13;12:434. doi: 10.1186/1471-2458-12-434 (PMC3487781; doi:10.1186/1471-2458-12-434)
Supplement: Additional file 2 — Table S2.Type 2 diabetes (T2DM) prevalence in Bangladesh: A summary of epidemiological studies published between1995 and 2010. [file 1471-2458-12-434-S2.doc]

**Additional Table 2** Type 2 diabetes (T2DM) prevalence in Bangladesh: A summary of epidemiological studies published between1995 and 2010.

| **Authors/Year** | **Diagnostic criteria** | **Sample Size** | **Sample Characteristics** | **Prevalence** | **Prevalence by Strata** | **Significant Risk Factors** |
| --- | --- | --- | --- | --- | --- | --- |
| Sayeed MA, 1995 [10] | FBG ≥ 7.0 and/or 2-hBG ≥ 11.0 | 1005 | rural  age > 15 | T2DM = 2.1% | male = 3.1%  female = 1.3% | age, BMI |
| Zaman MM, 2001 [14] | FBG ≥ 7.0 | 241 | rural  age ≥ 18 | T2DM=2.5% | male = 3.8%  female = 1.5% | not reported |
| Sayeed MA, 2003 [16] | FPG ≥ 7.0 | 4923 | rural  age > 20 | T2DM = 4.3% | male = 5.2%  female = 3.4% | age, BMI, income, family history,WHR, physical inactivity |
| Sayeed MA, 2005 [17] | FBG ≥ 6.0 and 2-hBG ≥ 7.8 | 147 | rural  pregnant women  age=18-44 | GDM:FBG= 6.8%, 2-hBG=8.2% | not applicable | not reported |
| Zaman MM, 2007 [20] | T2DM not defined | 447 | rural age ≥20 | T2DM=2.0% | not reported | not reported |
| Sayeed MA, 2007 [22] | FBG ≥ 6.1 and/or 2-hBG ≥ 11.1 | 5265 | urban  age > 20 | T2DM = 11.2% | slum = 7.4%non-slum = 13.4% | family history, income, sedentary lifestyle, age, BMI, WHR |
| Van Minh H,2008 [24] | self-reported | 8096 | rural  age = 25 - 64 | T2DM=12.0% | male = 11.2%  female = 12.7% | age, sex |
| Sayeed S, 2008 [25] | FBG ≥ 6.1 | 705 | urban  age ≥ 25 years | T2DM=21.1% | male = 27.1%  female = 18.0% | not reported |
| Ahsan SA, 2009 [27] | FBG ≥ 7.0 | 163 | urban  mean age = 44.8 | T2DM:12.3% | not reported | not reported |
| Das S, 2010 [28] | Unknown | 1200 | urban | T2DM:4.5% | unknown | Unknown |
| Sayeed MA, 1997 [30] | FBG ≥ 7.0 and/or 2-hBG ≥ 11.0 | 2371 | urban/rural  age > 20 | T2DM = 5.2% | male = 5.5%  female = 4.8%  urban= 8.0%  rural = 3.8% | age, sex, social class |
| Sayeed MA, 1997 [31] | 2-hBG ≥ 11.0 | 6847 | semi-urban  age ≥ 15 | T2DM = 4.0% | male = 4.1%  female = 3.9% | age, BMI, height |
| Rahim MA, 2004 [32] | FBG ≥ 6.1 | 1555 | urban slum  age > 20 | T2DM = 8.1% | male = 7.7%  female = 8.5% | age, sex, WHR |
| Sayeed MA, 2004 [33] | FPG ≥ 7.0 | 1287 | tribal  age > 20 | T2DM = 6.6% | male = 8.0%  female = 5.6% | age, income, WHR |
| Hussain, 2007 [34] | FBG > 6.1 | 4757 | rural  age > 20 | T2DM = 2.3% | male = 1.9%  female = 2.5% | Age, sex, high systolic blood pressure, WHR |
| Rahman MM, 2007 [35] | FBG > 6.1 | 975 | semi-rural  age > 20 | T2DM = 8.5% | male = 9.4%  female = 8.0% | age, BMI |
| Rahim MA, 2008 [36] | FBG ≥ 6.1or 2-hBG >11.0 | 3981 | rural  age > 20 | T2DM = 7.0% | male = 7.5%  female = 6.7% | not reported |

FBG: Fasting blood glucose, 2-hBG: 2 hour blood glucose, GDM: gestational diabetes mellitus, BMI: body mass index, WHR: waist to hip ratio
